# Use of Digital Tools and Environmental Practices Among Informal Caregivers of Older Adults in the European Union Embracing the Triple Transition: Cross-Sectional Study

**DOI:** 10.2196/81556

**Published:** 2026-11-13

**Authors:** Beatrice Ietto, Mirko Di Rosa, Giovanni Lamura, Sabrina Quattrini, Ricardo Rodrigues, Sara Santini, Aviad Tur-Sinai, Marco Socci

**Affiliations:** 1 Centre for Socio-Economic Research on Ageing, IRCCS INRCA, Ancona, Italy; 2 Centre for Biostatistics and Applied Geriatric Clinical Epidemiology, IRCCS INRCA, C.da Muoio Piccolo, Cosenza, 87100, Italy, 39 0718004604, 39 07135941; 3 ISEG Research, Lisbon School of Economics and Management, University of Lisbon, Lisbon, Portugal; 4 School of Public Health, The Faculty of Social Welfare and Health Sciences, University of Haifa, Haifa, Israel

**Keywords:** informal caregivers, digital tools, environmental behaviors, older adults, triple transition, European Union

## Abstract

**Background:**

The European Union is currently facing a demographic, digital, and environmental transition collectively known as the triple transition. Although these trends significantly impact long-term care services, they have typically been studied in isolation, overlooking potential synergies between the adoption of digital tools and environmental practices among informal caregivers of older adults. This study uniquely bridges this gap by applying an integrative social-ecological-infrastructural systems framework to examine how these transitions interact in everyday caregiving contexts.

**Objective:**

This study examined the relationship between the use of digital tools and eco-friendly practices among informal caregivers of older adults across the European Union and whether the adoption of digital solutions related differently to specific types of eco-friendly behaviors. Unlike previous research focusing on isolated aspects of each transition, we examined their interconnections, providing empirical evidence for developing integrated policy approaches.

**Methods:**

Using data from the Survey of Gender Gaps in Unpaid Care, Individual and Social Activities project (n=5761 informal caregivers of adults aged ≥65 years), we analyzed the association between frequency of use of digital tools for caregiving and environmental practices. We used the social-ecological-infrastructural systems conceptual framework and the theory of planned behavior (TPB) to interpret findings. Analyses included descriptive statistics, Spearman correlations, and multilevel mixed-effects regression models.

**Results:**

A weak positive association was observed between the frequency of use of digital tools and environmentally sustainable behaviors (ρ=0.28; *P*<.001). Among individual behaviors, the largest associations were observed for consumption-related behaviors (fair-trade products: ρ=0.30; used items: ρ=0.30; eco-friendly products: ρ=0.25), whereas the associations were weaker for waste management and transportation choices and were not statistically significant for recycling (ρ=−0.04; *P*=.23) and mindful consumption of natural resources (ρ=−0.02; *P*>.99). Respondents using digital tools daily had scores of environmentally sustainable behavior (SESBs) 22% higher than nonusers. Demographic patterns revealed higher use of digital tools among younger, more educated, and employed caregivers, indicating that any potential digital-environmental co-occurrence may be unevenly distributed across the caregiver population.

**Conclusions:**

The use of digital tools may be related to environmental behavior through improved access to information, peer norms, and a greater perceived ability to act, though these mechanisms were not directly tested. The stronger associations with consumption-related behaviors indicate that digital platforms may be particularly relevant to caregivers’ access to eco-friendly products, second-hand goods, fair-trade options, and sustainable local services. By contrast, the lack of statistically significant correlations with recycling and resource conservation suggests that digital solutions alone may be insufficient to address behaviors that depend on structural conditions, physical infrastructure, local service availability, or established household routines. Policies should consider digital inclusion, caregiver support, and green infrastructure as interconnected areas while ensuring that older, less educated, and more burdened caregivers are not excluded from these potential co-benefits.

## Introduction

### Setting the Context: The Triple Transition in Long-Term Care

The term “Triple Transition” has been used to describe the concomitant demographic, digital, and green transitions that the European Union (EU) aims to achieve through the integration of social inclusivity, technological advancement, and environmental sustainability [1]. According to the Organisation for Economic Co-operation and Development (OECD) [1], these transitions are reshaping societal structures, presenting unique challenges and opportunities in many sectors, including health care and, more specifically, long-term care (LTC) services.

In terms of demographic transition, the European LTC sector faces growing challenges such as rapid demographic aging and a shrinking working-age population. In Europe, median age increased from 39.0 to 44.5 years (2003‐2023), life expectancy increased by almost 4 years (2002‐2023), and the old-age dependency ratio grew by 9.3 percentage points (2001‐2020) [2,3]. These shifts might have serious implications for care provision, creating an unprecedented demand for care services together with a growing shortage of care staff [1,4].

The digital transition uses technology to enhance care delivery through electronic health records, telehealth, AI-powered diagnostics, and remote monitoring, among others [5,6]. Although some researchers predict digitalization will improve care efficiency [7], others caution that the benefits may be unequally distributed, causing a deeper digital exclusion and discrimination [1,8].

The green transition aims at fostering environmentally friendly practices as well as monitoring climate change [1]. As the health care sector contributes about 4.4% of global greenhouse gas emissions [9], care systems have faced increasing pressure to adopt more sustainable practices and policies [10,11].

While population aging is causing high pressure on the LTC sector and creating new challenges [12,13], digitalization and sustainability have been investigated as potential approaches to reduce resource constraints and improve the delivery of care [7,14-16].

In fact, while digital technologies have been primarily studied for enhancing care efficiency and quality [7,17-20], environmental practices seem to have been mainly investigated for their impact on resource efficiency, waste reduction, and management systems [21,22].

According to the OECD [1], these transitions have typically been studied in isolation, and potential synergies between digitalization and sustainability have been largely overlooked [23,24]. For example, digital solutions might simultaneously improve care efficiency as well as reduce environmental impact [25,26]. Similarly, LTC research has also maintained a siloed approach, with only a few studies exploring the integration and potential synergies between the digital and environmental transition [27-30]. Finally, Eurofound [3] calls for a more comprehensive understanding of these intersecting challenges, as this might inform solutions that jointly address rising care demand, technological change, and sustainability requirements in care systems.

### The Social-Ecological-Infrastructural-System Framework

To better understand the interconnections between the demographic, digital, and green transitions, this study uses the social-ecological-infrastructural systems (SEIS) framework developed by Ramaswami et al [31]. This framework belongs to a broader family of conceptual approaches that have been used to examine the relationships between the social, technological, and ecological dimensions of sustainability [32-34]. These approaches have been used to investigate how such dimensions are interconnected and to verify to what extent changes in one domain might affect the other ones [35,36]. Specifically, the SEIS framework merges 3 interconnected dimensions: social systems (people, communities, and institutions), infrastructural systems (transportation networks and engineered solutions), and ecological systems (natural environments and resources). Although the SEIS framework was originally conceived for urban sustainability [31], its application has been extended to other settings, including health care and sustainability [37]. More recent applications have also included digital technologies as a critical component of the infrastructural system [38,39]. Therefore, the SEIS framework is particularly suited to study the Triple Transition in LTC, as it directly aligns with the demographic (social), digital (infrastructural), and environmental (ecological) transitions occurring in the care sector.

The demographic transition primarily affects social systems through changes in population structure (ie, population aging), care needs, and the role of informal caregivers, for example, family or nonfamily members who provide unpaid care, outside of a formal framework, to someone with a chronic illness, disability, or other long-lasting care need [40]. The digital transition impacts the infrastructural system through the introduction of new tools, platforms, and devices for care delivery. Finally, the green transition seeks to develop more sustainable approaches for the use of resources and waste management.

While the Triple Transition has been viewed at these systems as separate pillars, the SEIS framework focuses on the integration within and among them. Specifically, it distinguishes 3 interfaces: the socioinfrastructural (how digital tools are integrated into caregiving contexts), the socioecological (how caregivers engage with eco-friendly practices), and the eco-infrastructural interface (how digital solutions contribute to environmental sustainability). The central intersection of the 3 systems represents the research gap addressed in this paper: the relationship between the use of digital tools and eco-friendly practices among informal caregivers (Figure 1).

**Figure 1.**
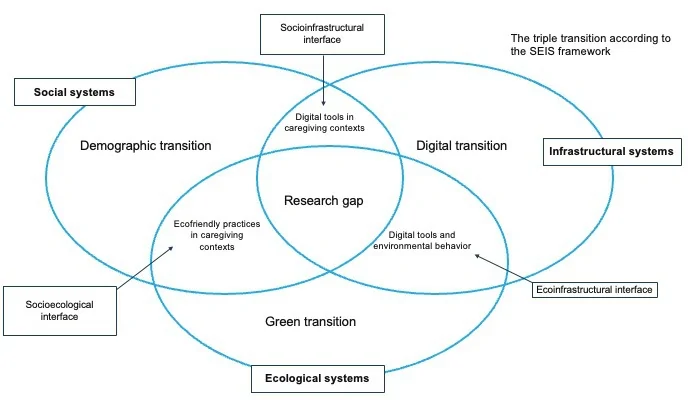
The Triple Transition according to the social-ecological-infrastructural systems (SEIS) framework (Ramaswami et al [31]). SEIS: social-ecological-infrastructural systems.

This study investigates how the use of digital tools is interconnected with the adoption of eco-friendly practices among informal caregivers. In doing so, it aims to better understand how the 3 dimensions of the SEIS framework interact with each other within the specific context of LTC. This integrated approach is consistent with more contemporary developments in sustainability theories that emphasize the need for systems thinking rather than siloed approaches [40].

Studying the intersection between informal caregivers’ use of digital tools and environmental practices provides a unique opportunity to understand how the Triple Transition manifests in care settings. Informal caregivers are a particularly important population group, increasingly affected by demographic shifts, digitalization, and growing environmental concerns. Situated at this intersection, they are especially relevant for understanding how these transitions interact in practice and how policy might better integrate these interconnected domains rather than addressing them in isolation. Drawing on the SEIS framework, the following 3 subsections examine the relationship between the use of digital tools and environmental behaviors; the adoption of digital tools in caregiving contexts; and eco-friendly practices in LTC.

### Digital Tools and Environmental Behaviors

The relationship between the adoption of digital technologies and environmental behaviors has gained increasing attention as both digitalization and environmental awareness have become more widespread [41,42]. This relationship is theoretically grounded in the concept of behavioral spillover effects [43], in which engagement in one domain (use of digital tools) may influence behaviors in another domain (environmental practices) through shifts in values, knowledge, and capabilities. In fact, digital tools may serve as enablers of environmental behaviors in a number of ways, including more efficient access to information, facilitating the creation of support communities, and making eco-friendly consumption easier to perform. According to Hilty and Aebischer [44], digital technology’s environmental effects can be categorized into 3 distinct orders.

First-order effects concern the direct environmental impacts of the life cycle of digital devices from production through use to disposal [44]. In the health care context, these impacts include the environmental footprint of digital and telehealth devices [27]. Second-order effects involve how digital technologies enable process optimization, dematerialization, and the substitution of physical resources with digital alternatives [44]. In health care and caregiving, digital services might reduce transportation emissions through telemedicine [45] and lower paper and other resource consumption through the adoption of electronic health records [22,46,47].

Third-order effects, which are considered to have the most profound impact, are the focus of this paper. They involve how digital technologies enable broader behavioral changes [44]. According to Santarius et al [48], digital technologies provide easier access to information about sustainable options, enable remote activities that reduce transportation needs, and foster the formation of online communities, in which peers share and discuss sustainable behaviors. Digital tools can also increase environmental awareness through educational content and real-time feedback [49,50].

Digital platforms make eco-friendly behaviors more convenient by reducing transaction costs of purchasing eco-friendly products [51-53]. Finally, online communities reinforce environmental norms and values through shared experiences and peer influence [54-56]. This explains how digital technologies might relate to environmental behaviors, even when digital tools themselves are not explicitly used for environmental purposes. While the understanding of digital tools’ environmental impact has grown, significant gaps remain in understanding how these effects manifest in specific populations such as caregivers. Research has primarily focused on first- and second-order impacts of digital technologies on environmental sustainability in health care [22,46]. The third-order behavioral effects, specifically how the use of digital tools relates to caregivers’ environmental behaviors, remain largely unexplored.

### Digital Tools in Caregiving Contexts

Digital tools have become increasingly integrated into caregiving practices, though adoption remains uneven. In the United States, approximately 53% of informal caregivers report using at least one digital tool for caregiving, although this varies significantly by tool type and demographic factors. For example, about 80% report feeling comfortable using basic technologies, compared with only 50% with advanced tools like smart assistants [57,58]. In other countries, where comparable data are scarce, digital solutions seem less widespread but increasingly being used to support informal caregiving [3,59,60]. In fact, they have been used for several purposes, including providing information and educational material [61], platforms for organizing caregiving activities [62], remote monitoring technologies [20], communities for social support [59,63], and administrative tools [64]. Despite variations in the use of digital tools across different caregivers’ activities, specialized caregiving apps have a relatively low adoption rate (25%) [65]. Furthermore, a significant digital divide still exists based on the caregivers’ demographic characteristics. For example, Shaffer et al [66] found that age and education are strong predictors of technology adoption, with 40.4% of caregivers aged 65 years and older identifying themselves as noninternet users versus just 13.7% of younger caregivers. The likelihood of being offline decreases by 40% among tertiary-educated caregivers, while rural caregivers show higher rates of digital exclusion [67]. The most critical barriers to adoption include financial constraints, digital literacy limitations, and privacy issues [64,67].

### Eco-Friendly Behaviors in LTC

The adoption of eco-friendly practices in LTC, particularly among informal caregivers, represents an emerging research area. Recent research has revealed that informal caregivers of older adults engage significantly more in environmentally sustainable behaviors compared to noncaregivers [68]. Their performance is particularly strong in sustainable consumption choices (eco-friendly products, fair-trade items, and second-hand goods), avoiding single-use plastics, prioritizing local food products, and using low-carbon transportation. While caregivers outperform noncaregivers across all measured environmental behaviors, the gap is somewhat smaller for household practices such as recycling and resource conservation [68].

This pattern suggests that caregivers’ environmental engagement extends beyond basic resource-saving behaviors to reflect deeper environmental values. The experience of caring for vulnerable individuals may foster a broader sense of responsibility and interconnectedness that extends to environmental concerns [69] and fosters a heightened ethic of care that embraces environmental stewardship [70,71]. Furthermore, because caregivers are typically in charge of households, they make several environmental decisions about consumption, waste disposal, and resource use [72]. This might foster an increased awareness of how environmental factors impact an individual’s health, leading to the adoption of a long-term consciousness of the importance of sustainability for future generations [73].

Nonetheless, the distinctive constraints that caregivers face, including time limitations [74,75], financial pressures often associated with longer hours of care [76], and mental and physical exhaustion [76,77], could reduce their engagement in environmental practices. Understanding how digital tools might help overcome these barriers is particularly relevant to supporting sustainable behaviors among this substantial population group, which already shows a predisposition toward eco-friendly practices [75,78,79].

### Research Questions

This study offers practical insights into potential interventions that could simultaneously promote digital inclusion and support environmentally sustainable behaviors among informal caregivers, a group that often faces considerable time limitations, financial pressures, and emotional demands. By empirically examining the relationship between the use of digital tools and eco-friendly practices in caregiving contexts, the study addresses a critical gap in the existing literature, which has largely considered digital and environmental transitions in isolation. In doing so, it contributes to a more integrated understanding of how the digital and environmental transitions intersect in everyday care and may inform policies that treat the 2 domains together rather than in isolation. Accordingly, this study addresses 2 key research questions:

1. What is the relationship between the use of digital tools and eco-friendly practices among informal caregivers of older adults with LTC needs?
2. Does the use of digital tools interlace differently with some eco-friendly practices rather than others?

Beyond estimating whether the use of digital tools is associated with environmentally sustainable behaviors, the analysis is intended to identify the behaviors for which digital interventions may be most relevant and those for which they are likely to be of limited use.

The focus on informal caregivers of older adults with LTC needs builds on previous research [68], which suggests that this population shows distinct patterns of environmental engagement compared to noncaregivers. These caregivers face unique challenges at the intersection of aging demographics, digital adoption barriers, and environmental concerns. As the population continues to age in Europe, understanding how they navigate both the digital and environmental transitions is increasingly relevant for the development of sustainable LTC policy.

### Theoretical Approach

To examine how the use of digital tools relates to environmental practices among informal caregivers of older adults, this study uses the theory of planned behavior (TPB) [80] as its primary analytical framework. TPB offers a well-established psychological model of the behavioral determinants, making it particularly suitable for exploring how digital tools might be associated with caregivers’ environmental behaviors. According to this theory, behavioral intentions are determined by 3 key factors: attitudes (evaluations of the behavior), subjective norms (perceived social pressure), and perceived behavioral control (perceived ease or difficulty of performing a behavior).

The application of TPB is especially relevant for informal caregivers who face distinctive pressures and motivations compared with those of the general population. Caregivers often operate under significant time pressures, financial constraints, and emotional burdens that may influence their environmental practices. Through the TPB lens, we can examine how digital tools might affect each component in the caregiving context:

- Attitudes: digital tools may influence caregivers’ evaluations of eco-friendly practices by providing information and educational content about environmental issues and sustainable practices [49,50]. Online resources can expose caregivers to environmental information that shapes their judgments about these practices
- Subjective norms: digital communities and social networks can strengthen environmental norms among caregivers [55,56], potentially normalizing eco-friendly behaviors or creating social pressure to meet expectations for environmental responsibility
- Perceived behavioral control: digital tools may enhance caregivers’ perceived ability to engage in environmental behaviors by making sustainable options more accessible or convenient, for example, through e-commerce platforms [51-53]. This effect is likely to be more pronounced for behaviors that can be changed through information or convenience than for those dependent on external infrastructure

Building on recent extensions of the TPB [80,81], sociodemographic factors that may be associated with these relationships are also considered. Age, education, employment status, urbanization, and country of residence may be systematically related to both the adoption of digital tools and environmental behaviors. Previous research indicates that younger age [66], higher education, and urban residence [67] are associated with more positive attitudes toward digital technologies, potentially contributing to variation in the relationship between digital tools and environmental behaviors across different caregiver subgroups. This approach aligns with the SEIS conceptual framework by providing a psychological dimension for understanding how social (caregiving context), infrastructural (digital tools), and ecological (environmental behaviors) systems interact at the individual level. The analyzed eco-friendly behaviors include consumption choices (eco-friendly products, fair-trade items, second-hand goods, or plant-based options), resource conservation, waste management practices, and transportation decisions [82]. By examining how the use of digital tools relates to these different behavior categories, we can identify which TPB components may best account for the observed associations in the informal caregiving context.

## Methods

### Main Study Design and Participants

The findings outlined in this study are based on the Survey of Gender Gaps in Unpaid Care, Individual, and Social Activities project (European Institute for Gender Equality; EIGE/2021/OPER/10)—commissioned by the EIGE—conducted between November 2021 and December 2022 by Eurocarers (Belgium), in collaboration with the European Centre for Social Welfare Policy and Research (Austria), the National Institute of Health and Science on Ageing (Istituto di Ricovero e Cura a Carattere Scientifico [IRCCS] Istituto Nazionale Ricovero e Cura Anziani [INRCA] Italy), and IPSOS GmbH (Germany). In total, 60,405 individuals aged 16-74 years participated in the survey, which was carried out across all 27 EU Member States using quantitative methods. The fieldwork took place between August and October 2022.

### Sampling and Data Collection

The primary data-collection instrument was a questionnaire designed for a 15-minute survey and structured to comply with the core principles of the General Data Protection Regulation (EU) 2018/1725 (GDPR). Data were collected by IPSOS GmbH using computer-assisted web interviews (CAWIs) across all participating countries except Malta and Luxembourg, where interviews were conducted via computer-assisted telephone interviews (CATIs). For the CAWI mode, the target population was limited to individuals who had registered with an online access panel, who were residents of one of the Member States, and who had access to online services on a digital device at the time of the survey. Pane­ls are pools of registered respondents who are willing to participate in market and opinion research. To register, respondents must reside in the country of the panel they are signing up for. Their language skills must be sufficient to answer the registration questions and surveys in the local language. Robust online access panels do not exist in all EU Member States, particularly in smaller countries, where the overall size of such panels is limited. In Luxembourg and Malta, the available panels could not guarantee a sufficient number of respondents to reach the national sample sizes established on the sampling design. Therefore, in these 2 countries, respondents were interviewed via CATIs [68,83].

The sample size varied by country according to population size, ranging from 500 respondents for smaller countries (eg, Cyprus) to 4000 for more populous countries (eg, France and Germany). Hard quotas were applied for age, gender, and region (Nomenclature of Territorial Units for Statistics–Level 3 [NUTS III] classification), while monitoring quotas covered factors such as household composition, number of children, degree of urbanization, and education level (according to International Standard Classification of Education [ISCED] level of low, medium, and high) [68]. Monitoring education in particular helped account for differing levels of digital literacy among respondents, mitigating potential bias in the CAWI sample.

The survey targeted the general population of individuals aged 16-74 years. In certain countries (Austria, Belgium, Spain, Lithuania, Luxembourg, Latvia, Malta, and Poland), a limited number of respondents aged 75 years and older were included in the survey to help meet quotas for the 65-74-year age group. In Romania, constraints related to low internet accessibility and limited digital literacy led to a narrower age range, restricting participation to individuals aged 16-64 years. The target quotas were based on official Eurostat statistics and reflected the population stratification within each Member State. The sampling approach for the CATIs differed from that for the CAWIs: no quotas were applied in Luxembourg or Malta, because the sampling was random, thereby reducing the potential for selection bias. Any discrepancies between the net sample and representative figures were subsequently adjusted through weighting. A random iterative proportional fitting procedure was used to weight multiple variables so that, after a predefined maximum number of iterations, the weighted distribution matched the target values of all selected variables [84].

### Sample Selection: Exclusion Criteria

The total EIGE project sample numbered 60,405 participants. The exclusion criteria for this study were as follows:

- Participants who did not provide informal care: 47,209
- Participants whose main informal care recipient was younger than 65 years: 6273
- Participants with missing data on the frequency of sustainable behaviors: 1068
- Participants with missing data on the frequency of digital tool use in their caregiving roles: 94.

The resulting subsample selected for this paper, after the above exclusion criteria were applied, comprised 5761 individuals, who were included in the following analysis.

### Outcomes and Dimensions

#### Outcome Variables

The outcome variable of the study was a score of environmentally sustainable behavior (SESB), calculated from participants’ answers to 10 questions referring to environmentally friendly behaviors (see Multimedia Appendix 1 for the complete list of these 10 questions). For validity, internal consistency, and further details of the score, see Tur-Sinai et al [68]. To build the score, questions were coded from 1 (“Never”) to 5 (“Daily”) and summed to obtain a score ranging from 10 to 50 (higher scores indicated more frequent environmentally sustainable behaviors).

#### Covariates

The demographic covariates included gender and age. Socioeconomic variables included highest educational level according to the ISCED (2011; low=ISCED 0‐2; medium=ISCED 3‐4; high=ISCED 5+), employment status at the time of the survey (employed, unemployed, retired, or other nonworking situation), and perceived adequacy of household income (with great difficulty, with difficulty, with some difficulty, fairly easily, easily, or very easily). Weekly hours of informal care were assessed via 6-hour bands (1‐7 hours, 8-21 hours, 22‐35 hours, 36-39 hours, 50‐70 hours, and ≥71 hours). We also included information about the main form of residence (in your household, in another household, or in a care institution full-time or part-time) and the degree of urbanization of the place of residence (densely populated area, intermediate density area, and sparsely populated area) and country of residence. Finally, we included self-assessed general health (very good, good, fair, bad, or very bad).

### Statistical Analysis

For descriptive statistics, the distribution normality of continuous variables was assessed using the Shapiro-Wilk test; means and SDs were reported. Statistical significance of between-group comparisons was assessed using one-way analysis of variance. For categorical variables, absolute frequencies and percentages were reported, and statistical significance was assessed using the Pearson chi-square test. The relationship between the frequency of use of digital tools in the caregiving role and the overall SESB score and each individual environmentally sustainable behavior was evaluated using Spearman rank correlation coefficients, with Bonferroni-adjusted *P* values.

For the multivariate analyses, a linear mixed effects model using maximum likelihood estimation was applied to the SESB score, while 10 mixed effects ordered probit models were estimated for the individual environmentally sustainable behaviors. Because of the hierarchical nature of the data, the statistical approach needed to account for the nesting of individuals within countries [85]. To address this, multilevel mixed effects models using maximum likelihood were employed, enabling the total variability to be partitioned into 2 levels: the primary level (individual-specific variability) and the secondary level (country-level variability) [84].

The validity of the models was evaluated using the Wald chi-square test. Additionally, to identify potential multicollinearity issues among covariates, the standard errors of each coefficient were assessed to mitigate variance inflation.

A 2-tailed *P* value of <.05 was considered statistically significant. All data analyses were conducted using STATA/MP (version 19.0; StataCorp LLC).

The survey microdata are publicly available on the GESIS public repository [86].

### Ethical Considerations

The study adhered to the ethical principles outlined in the Declaration of Helsinki, and the survey protocol (including ethical aspects) received approval from IPSOS’ Global Data Privacy and Research Compliance Team [87]. Some modifications to the questionnaire were required before approval, including the inclusion of explicit consent for sensitive data, the addition of refusal options, and the use of bracketed/grouped responses when referring to personal details about another adult individual (ie, the partner).

The survey was conducted in full compliance with national and EU data protection regulations, including the GDPR (Regulation [EU] 2016/679), Regulation [EU] 2018/1725, and Regulation [EC] No. 223/2009 regarding the processing of data for statistical aims. An integrated EU-27 dataset was generated from anonymized national datasets, ensuring that respondents could not be identified. A separate informed-consent clause was embedded in the survey’s introduction, informing participants of their rights regarding data access, rectification, withdrawal, and restriction, as well as their right to object to data processing and to file a complaint with the relevant data protection supervisory authority.

Respondents did not receive any compensation for participating in the survey. Informed consent was obtained from all participants, as the survey included questions related to political engagement and health. Respondents were provided with a dedicated section explaining the survey’s purpose, content, and data storage policies. Only after giving their consent were they allowed to participate in the survey.

## Results

Table 1 presents a bivariate analysis of the relationship between the frequency of use of digital tools for care-related activities and two sets of variables: (1) environmentally sustainable behaviors among informal caregivers—including recycling, avoiding single-use plastics, purchasing eco-friendly and fair-trade products, buying second-hand items, choosing plant-based foods, prioritizing local/seasonal food, mindful resource use, and low-carbon transportation—and (2) sociodemographic covariates. The analysis reveals a consistent positive association between the frequency of use of digital tools and environmentally sustainable behaviors. Specifically, caregivers who use digital tools daily have SESB scores 22% higher than nonusers, with a mean difference of 7 points. This indicates that a more frequent use of digital tools is associated with greater engagement in eco-friendly caregiving practices.

**Table 1.** Sample description according to the frequency of use of digital tools and of environmentally sustainable behaviors as a caregiver.

Frequency use of digital tools	Total (n=5761)	Never (n=1616)	Less often (n=1446)	Several times a month (n=864)	Several times a week (n=851)	Daily (n=984)	*P* value
Frequency of sustainable behavior							
SESB^a^ (10-50), mean (SD)	33.9 (8.3)	31.4 (8.2)	32.9 (7.3)	33.3 (7.5)	36.0 (7.3)	38.4 (9.0)	<.001
Avoidance of plastic and/or disposable/single-use products, n(%)							<.001
Never	379 (6.6)	184 (11.4)	70 (4.8)	45 (5.2)	23 (2.7)	57 (5.8)	
Less often	1125 (19.5)	392 (24.3)	326 (22.5)	159 (18.4)	122 (14.3)	126 (12.8)	
Several times a month	1263 (21.9)	344 (21.3)	362 (25.0)	236 (27.3)	182 (21.4)	139 (14.1)	
Several times a week	1449 (25.2)	326 (20.2)	362 (25.0)	235 (27.2)	281 (33.0)	245 (24.9)	
Daily	1545 (26.8)	370 (22.9)	326 (22.5)	189 (21.9)	243 (28.6)	417 (42.4)	
Recycling, n (%)							<.001
Never	170 (3)	71 (4.4)	35 (2.4)	22 (2.5)	16 (1.9)	26 (2.6)	
Less often	545 (9.5)	165 (10.2)	142 (9.8)	104 (12.0)	63 (7.4)	71 (7.2)	
Several times a month	773 (13.4)	149 (9.2)	212 (14.7)	155 (17.9)	149 (17.5)	108 (11.0)	
Several times a week	1062 (18.4)	215 (13.3)	246 (17)	184 (21.3)	218 (25.6)	199 (20.2)	
Daily	3211 (55.7)	1016 (62.9)	811 (56.1)	399 (46.2)	405 (47.6)	580 (58.9)	
Avoidance of animal products, n (%)							<.001
Never	1493 (25.9)	622 (38.5)	396 (27.4)	175 (20.3)	132 (15.5)	168 (17.1)	
Less often	1488 (25.8)	424 (26.2)	485 (33.5)	231 (26.7)	177 (20.8)	171 (17.4)	
Several times a month	1056 (18.3)	237 (14.7)	266 (18.4)	202 (23.4)	207 (24.3)	144 (14.6)	
Several times a week	1015 (17.6)	197 (12.2)	189 (13.1)	184 (21.3)	229 (26.9)	216 (22.0)	
Daily	709 (12.3)	136 (8.4)	110 (7.6)	72 (8.3)	106 (12.5)	285 (29.0)	
Low-carbon-emission modes of transport, n (%)							<.001
Never	844 (14.7)	400 (24.8)	180 (12.4)	96 (11.1)	71 (8.3)	97 (9.9)	
Less often	1314 (22.8)	401 (24.8)	406 (28.1)	217 (25.1)	149 (17.5)	141 (14.3)	
Several times a month	1001 (17.4)	223 (13.8)	276 (19.1)	198 (22.9)	170 (20.0)	134 (13.6)	
Several times a week	1229 (21.3)	252 (15.6)	284 (19.6)	216 (25)	261 (30.7)	216 (22.0)	
Daily	1373 (23.8)	340 (21.0)	300 (20.7)	137 (15.9)	200 (23.5)	396 (40.2)	
Buy eco-friendly products and services, n (%)							<.001
Never	302 (5.2)	145 (9.0)	58 (4.0)	35 (4.1)	17 (2.0)	47 (4.8)	
Less often	1278 (22.2)	478 (29.6)	371 (25.7)	173 (20)	126 (14.8)	130 (13.2)	
Several times a month	1617 (28.1)	445 (27.5)	485 (33.5)	286 (33.1)	232 (27.3)	169 (17.2)	
Several times a week	1422 (24.7)	333 (20.6)	313 (21.6)	242 (28.0)	285 (33.5)	249 (25.3)	
Daily	1142 (19.8)	215 (13.3)	219 (15.1)	128 (14.8)	191 (22.4)	389 (39.5)	
Buy fair-trade products and services, n (%)							<.001
Never	496 (8.6)	259 (16.0)	93 (6.4)	56 (6.5)	31 (3.6)	57 (5.8)	
Less often	1503 (26.1)	540 (33.4)	441 (30.5)	204 (23.6)	175 (20.6)	143 (14.5)	
Several times a month	1494 (25.9)	404 (25.0)	443 (30.6)	279 (32.3)	209 (24.6)	159 (16.2)	
Several times a week	1235 (21.4)	235 (14.5)	300 (20.7)	224 (25.9)	242 (28.4)	234 (23.8)	
Daily	1033 (17.9)	178 (11.0)	169 (11.7)	101 (11.7)	194 (22.8)	391 (39.7)	
Buy used instead of new items, n (%)							<.001
Never	1106 (19.2)	474 (29.3)	267 (18.5)	133 (15.4)	95 (11.2)	137 (13.9)	
Less often	2025 (35.2)	671 (41.5)	625 (43.2)	272 (31.5)	236 (27.7)	221 (22.5)	
Several times a month	1170 (20.3)	248 (15.3)	317 (21.9)	244 (28.2)	197 (23.1)	164 (16.7)	
Several times a week	754 (13.1)	104 (6.4)	133 (9.2)	154 (17.8)	205 (24.1)	158 (16.1)	
Daily	706 (12.3)	119 (7.4)	104 (7.2)	61 (7.1)	118 (13.9)	304 (30.9)	
Prioritize seasonal and local food products, n (%)							<.001
Never	199 (3.5)	89 (5.5)	38 (2.6)	25 (2.9)	19 (2.2)	28 (2.8)	
Less often	713 (12.4)	240 (14.9)	206 (14.2)	113 (13.1)	72 (8.5)	82 (8.3)	
Several times a month	1281 (22.2)	362 (22.4)	362 (25.0)	236 (27.3)	193 (22.7)	128 (13.0)	
Several times a week	1649 (28.6)	426 (26.4)	409 (28.3)	286 (33.1)	288 (33.8)	240 (24.4)	
Daily	1919 (33.3)	499 (30.9)	431 (29.8)	204 (23.6)	279 (32.8)	506 (51.4)	
Mindful of recyclability or biodegradability of products and packaging, n (%)							<.001
Never	453 (7.9)	231 (14.3)	95 (6.6)	50 (5.8)	28 (3.3)	49 (5)	
Less often	1046 (18.2)	361 (22.3)	304 (21)	159 (18.4)	113 (13.3)	109 (11.1)	
Several times a month	1160 (20.1)	305 (18.9)	332 (23)	205 (23.7)	169 (19.9)	149 (15.1)	
Several times a week	1416 (24.6)	306 (18.9)	351 (24.3)	255 (29.5)	296 (34.8)	208 (21.1)	
Daily	1686 (29.3)	413 (25.6)	364 (25.2)	195 (22.6)	245 (28.8)	469 (47.7)	
Mindful consumption of natural resources, n (%)							<.001
Never	159 (2.8)	72 (4.5)	28 (1.9)	26 (3.0)	15 (1.8)	18 (1.8)	
Less often	464 (8.1)	126 (7.8)	125 (8.6)	89 (10.3)	58 (6.8)	66 (6.7)	
Several times a month	812 (14.1)	182 (11.3)	219 (15.1)	168 (19.4)	137 (16.1)	106 (10.8)	
Several times a week	1130 (19.6)	222 (13.7)	308 (21.3)	195 (22.6)	229 (26.9)	176 (17.9)	
Daily	3196 (55.5)	1014 (62.7)	766 (53.0)	386 (44.7)	412 (48.4)	618 (62.8)	
Covariates							
Sex, n (%)							<.001
Female	3155 (54.8)	987 (61.1)	763 (52.8)	407 (47.1)	459 (53.9)	539 (54.8)	
Male	2591 (45.0)	624 (38.6)	681 (47.1)	457 (52.9)	388 (45.6)	441 (44.8)	
Other	15 (0.3)	5 (0.3)	2 (0.1)	0 (0)	4 (0.5)	4 (0.4)	
Age, mean (SD)	46.9 (14.0)	50.4 (13.2)	48.1 (13.7)	45.4 (14.4)	43.3 (13.8)	43.9 (14.0)	<.001
Education level, n (%)							<.001
Low	599 (10.4)	227 (14.0)	116 (8.0)	78 (9.0)	79 (9.3)	99 (10.1)	
Medium	2376 (41.2)	676 (41.8)	615 (42.5)	344 (39.8)	332 (39)	409 (41.6)	
High	2786 (48.4)	713 (44.1)	715 (49.4)	442 (51.2)	440 (51.7)	476 (48.4)	
Degree of urbanization							.02
Densely populated area	2685 (46.6)	696 (43.1)	670 (46.3)	423 (49.0)	421 (49.5)	475 (48.3)	
Intermediate density area	1959 (34)	589 (36.4)	512 (35.4)	284 (32.9)	265 (31.1)	309 (31.4)	
Sparsely populated area	1117 (19.4)	331 (20.5)	264 (18.3)	157 (18.2)	165 (19.4)	200 (20.3)	
Perceived household income: household is able to make ends meet, n (%)							<.001
With great difficulty	407 (7.1)	117 (7.2)	71 (4.9)	50 (5.8)	56 (6.6)	113 (11.5)	
With difficulty	692 (12)	177 (11)	165 (11.4)	95 (11)	108 (12.7)	147 (14.9)	
With some difficulty	1719 (29.8)	437 (27)	461 (31.9)	265 (30.7)	270 (31.7)	286 (29.1)	
Fairly easily	1206 (20.9)	340 (21)	288 (19.9)	196 (22.7)	199 (23.4)	183 (18.6)	
Easily	628 (10.9)	167 (10.3)	167 (11.5)	106 (12.3)	87 (10.2)	101 (10.3)	
Very easily	272 (4.7)	68 (4.2)	76 (5.3)	43 (5.0)	39 (4.6)	46 (4.7)	
Prefer not to answer	837 (14.5)	310 (19.2)	218 (15.1)	109 (12.6)	92 (10.8)	108 (11)	
Current situation, n (%)							<.001
Employed	3827 (66.4)	976 (60.4)	968 (66.9)	604 (69.9)	594 (69.8)	685 (69.6)	
Unemployed	351 (6.1)	111 (6.9)	69 (4.8)	44 (5.1)	66 (7.8)	61 (6.2)	
Retired	755 (13.1)	271 (16.8)	222 (15.4)	99 (11.5)	56 (6.6)	107 (10.9)	
Other nonworking situation	828 (14.4)	258 (16.0)	187 (12.9)	117 (13.5)	135 (15.9)	131 (13.3)	
Weekly hours of informal care for main care recipient, mean (SD)	2.0 (1.3)	1.9 (1.2)	1.8 (1.1)	2.1 (1.2)	2.1 (1.2)	2.4 (1.5)	<.001
Residence of main recipient of informal care during period of caring, n (%)							<.001
In your household	1447 (25.1)	406 (25.1)	310 (21.4)	201 (23.3)	215 (25.3)	315 (32)	
In another household	3609 (62.6)	1022 (63.2)	973 (67.3)	552 (63.9)	524 (61.6)	538 (54.7)	
In a care institution	656 (11.4)	172 (10.6)	156 (10.8)	101 (11.7)	104 (12.2)	123 (12.5)	
Prefer not to answer	49 (0.9)	16 (1.0)	7 (0.5)	10 (1.2)	8 (0.9)	8 (0.8)	
General health, n (%)							<.001
Very good	958 (16.6)	219 (13.6)	218 (15.1)	138 (16.0)	162 (19.0)	221 (22.5)	
Good	2544 (44.2)	685 (42.4)	678 (46.9)	411 (47.6)	390 (45.8)	380 (38.6)	
Fair (neither good nor bad)	1814 (31.5)	558 (34.5)	450 (31.1)	261 (30.2)	244 (28.7)	301 (30.6)	
Bad	366 (6.4)	125 (7.7)	91 (6.3)	46 (5.3)	41 (4.8)	63 (6.4)	
Very bad	52 (0.9)	20 (1.2)	6 (0.4)	5 (0.6)	9 (1.1)	12 (1.2)	
Prefer not to answer	27 (0.5)	9 (0.6)	3 (0.2)	3 (0.3)	5 (0.6)	7 (0.7)	
Country, n (%)							<.001
Ireland	85 (1.5)	27 (1.7)	17 (1.2)	12 (1.4)	12 (1.4)	17 (1.7)	
Austria	136 (2.4)	51 (3.2)	29 (2.0)	16 (1.9)	16 (1.9)	24 (2.4)	
Belgium	232 (4.0)	105 (6.5)	53 (3.7)	35 (4.1)	16 (1.9)	23 (2.3)	
Bulgaria	257 (4.5)	51 (3.2)	56 (3.9)	35 (4.1)	44 (5.2)	71 (7.2)	
Croatia	204 (3.5)	41 (2.5)	71 (4.9)	30 (3.5)	35 (4.1)	27 (2.7)	
Cyprus	37 (0.6)	11 (0.7)	14 (1.0)	2 (0.2)	6 (0.7)	4 (0.4)	
Czechia	209 (3.6)	84 (5.2)	54 (3.7)	30 (3.5)	23 (2.7)	18 (1.8)	
Denmark	103 (1.8)	20 (1.2)	18 (1.2)	20 (2.3)	23 (2.7)	22 (2.2)	
Estonia	43 (0.7)	11 (0.7)	18 (1.2)	2 (0.2)	6 (0.7)	6 (0.6)	
Finland	148 (2.6)	40 (2.5)	45 (3.1)	15 (1.7)	30 (3.5)	18 (1.8)	
France	247 (4.3)	89 (5.5)	77 (5.3)	29 (3.4)	28 (3.3)	24 (2.4)	
Germany	306 (5.3)	124 (7.7)	51 (3.5)	45 (5.2)	50 (5.9)	36 (3.7)	
Greece	438 (7.6)	90 (5.6)	112 (7.7)	69 (8.0)	83 (9.8)	84 (8.5)	
Hungary	172 (3.0)	33 (2.0)	36 (2.5)	36 (4.2)	26 (3.1)	41 (4.2)	
Italy	567 (9.8)	204 (12.6)	122 (8.4)	89 (10.3)	74 (8.7)	78 (7.9)	
Latvia	62 (1.1)	15 (0.9)	15 (1.0)	9 (1.0)	10 (1.2)	13 (1.3)	
Lithuania	129 (2.2)	29 (1.8)	36 (2.5)	23 (2.7)	15 (1.8)	26 (2.6)	
Netherlands	286 (5.0)	127 (7.9)	61 (4.2)	37 (4.3)	27 (3.2)	34 (3.5)	
Poland	406 (7.0)	80 (5.0)	108 (7.5)	64 (7.4)	65 (7.6)	89 (9.0)	
Portugal	402 (7.0)	100 (6.2)	108 (7.5)	62 (7.2)	58 (6.8)	74 (7.5)	
Romania	408 (7.1)	68 (4.2)	104 (7.2)	57 (6.6)	62 (7.3)	117 (11.9)	
Slovakia	70 (1.2)	19 (1.2)	30 (2.1)	8 (0.9)	8 (0.9)	5 (0.5)	
Slovenia	106 (1.8)	29 (1.8)	31 (2.1)	14 (1.6)	17 (2.0)	15 (1.5)	
Spain	509 (8.8)	110 (6.8)	117 (8.1)	95 (11.0)	93 (10.9)	94 (9.6)	
Sweden	142 (2.5)	33 (2.0)	49 (3.4)	20 (2.3)	21 (2.5)	19 (1.9)	
Luxembourg	24 (0.4)	6 (0.4)	9 (0.6)	5 (0.6)	2 (0.2)	2 (0.2)	
Malta	33 (0.6)	19 (1.2)	5 (0.3)	5 (0.6)	1 (0.1)	3 (0.3)	

a SESB: score of environmentally sustainable behavior.

Interestingly, while all 10 environmental behaviors included in the SESB are statistically significant, they show different patterns. For 8 behaviors, the proportion of caregivers engaging in daily eco-friendly practices is progressively higher from nonusers to daily digital users. Two behaviors, however, deviate from this pattern. For “recycling” and “mindful consumption of natural resources,” engagement does not rise steadily with the frequency of use of digital tools. It remains relatively stable across groups and, in the case of recycling, it declines among the heaviest users. In both cases, moderate digital users show the lowest engagement, producing a U-shaped distribution across the 5 frequency groups.

When considering each eco-friendly behavior separately, Table 1 shows the strongest relationship between the use of digital tools and consumption-related behaviors. Daily users of digital tools are almost 4 times more likely to buy fair-trade products daily (39.7%) than never-users (11%). Conversely, the percentage of caregivers who never buy fair-trade products falls from 16% among never-users of digital tools to 5.8% among daily users.

Daily users of digital tools are over 4 times more likely to purchase used items daily (30.9%) than never-users (7.4%). Conversely, never-users are more than twice as likely to never buy used items (29.3%) than daily users (13.9%).

Daily users of digital tools are 3 times more likely to purchase eco-friendly products daily (39.5%) compared to never-users (13.3%). Never-users are almost twice as likely to never buy eco-friendly products (9.0%) than daily users (4.8%).

Users of digital tools are over 3 times more likely to consume plant-based products (avoid animal products) daily than never-users. Conversely, never-users are more than twice as likely to never avoid animal products (38.5%) than daily users (17.1%).

In terms of resource conservation and waste management, daily users of digital tools are almost twice as likely to avoid single-use plastics daily (42.4%) than never-users (22.9%) and show much higher rates of daily prioritization of seasonal/local food (51.4%) than never-users (30.9%). Daily users of digital tools are much more likely to consider product recyclability daily (47.7%) than never-users (25.6%).

In terms of transportation decisions, daily users of digital tools are almost twice as likely to use low-carbon transportation daily (40.2%) than never-users (21.0%). Conversely, never-users are 2.5 times more likely to never use low-carbon transportation (24.8%) than daily users (9.9%). As for frequency of use of digital tools for care-related activities and sociodemographic covariates, the analysis suggests some specific patterns in the adoption of digital tools (Table 1). In terms of age, never users of digital tools are the oldest group (mean 50.4, SD 13.2 years), while daily users are younger (mean 43.9, SD 14.0 years). Higher education is also associated with more frequent use of digital tools. Daily users have higher rates of high education (48.4%) compared to never users (44.1%). Never users have higher rates of low education (14.0%) compared to daily users (10.1%). Daily users are much more likely to be employed (69.6%) than never users (60.4%), while retired individuals are significantly less likely to use digital tools on a daily basis (10.9%) compared to never users (16.8%). There is a slightly higher use of digital tools in densely populated areas (48.3% of daily users vs 43.1% of never-users). However, the difference in the adoption of digital tools is minimal in scarcely populated areas (20.3% vs 20.5%).

The peculiarity of recycling and mindful consumption of natural resources is further confirmed by the correlation analysis in Table 2. The frequency of use of digital tools shows a weak positive correlation with overall environmental behaviors (ρ=0.28; *P*<.001), with the largest associations observed for consumption-related behaviors including fair-trade products (ρ=0.30), used items (ρ=0.30), and eco-friendly products (ρ=0.25). In contrast, the weakest relationships are found for recycling (ρ=−0.04; *P*=.22) and mindful consumption of natural resources (ρ=−0.02; *P*>.99), neither of which shows a statistically significant correlation with the frequency of use of digital tools among informal caregivers.

**Table 2.** Spearman rank correlation coefficients with Bonferroni-adjusted *P* values between environmentally sustainable behaviors and frequency of use of digital tools for the role of carer.

Frequency of sustainable behavior	ρ	*P* value
SESB^a^	0.28	<.001
Avoidance of plastic and/or disposable/single-use products	0.18	<.001
Recycling	–0.04	.23
Avoidance of animal products	0.27	<.001
Use of low-carbon-emission modes of transport	0.20	<.001
Purchase of eco-friendly products and services	0.25	<.001
Purchase of fair-trade products and services	0.30	<.001
Purchase of used (instead of new) items	0.30	<.001
Prioritization of seasonal and local food products	0.14	<.001
Mindful of recyclability or biodegradability of products and packaging	0.20	<.001
Mindful of consumption of natural resources	–0.02	>.99

a SESB: score of environmentally sustainable behavior.

Figure 2 shows how countries score on average for frequency of use of digital tools and of environmentally sustainable behaviors. Interestingly, countries where caregivers report higher scores on both dimensions are almost all located in Eastern Europe (eg, Poland, Romania, and Bulgaria) and Southern Europe (eg, Portugal and Spain). At the opposite end sit the Benelux countries (Belgium, Luxembourg, and the Netherlands), with some outliers such as Malta, Cyprus, and Czechia.

**Figure 2.**
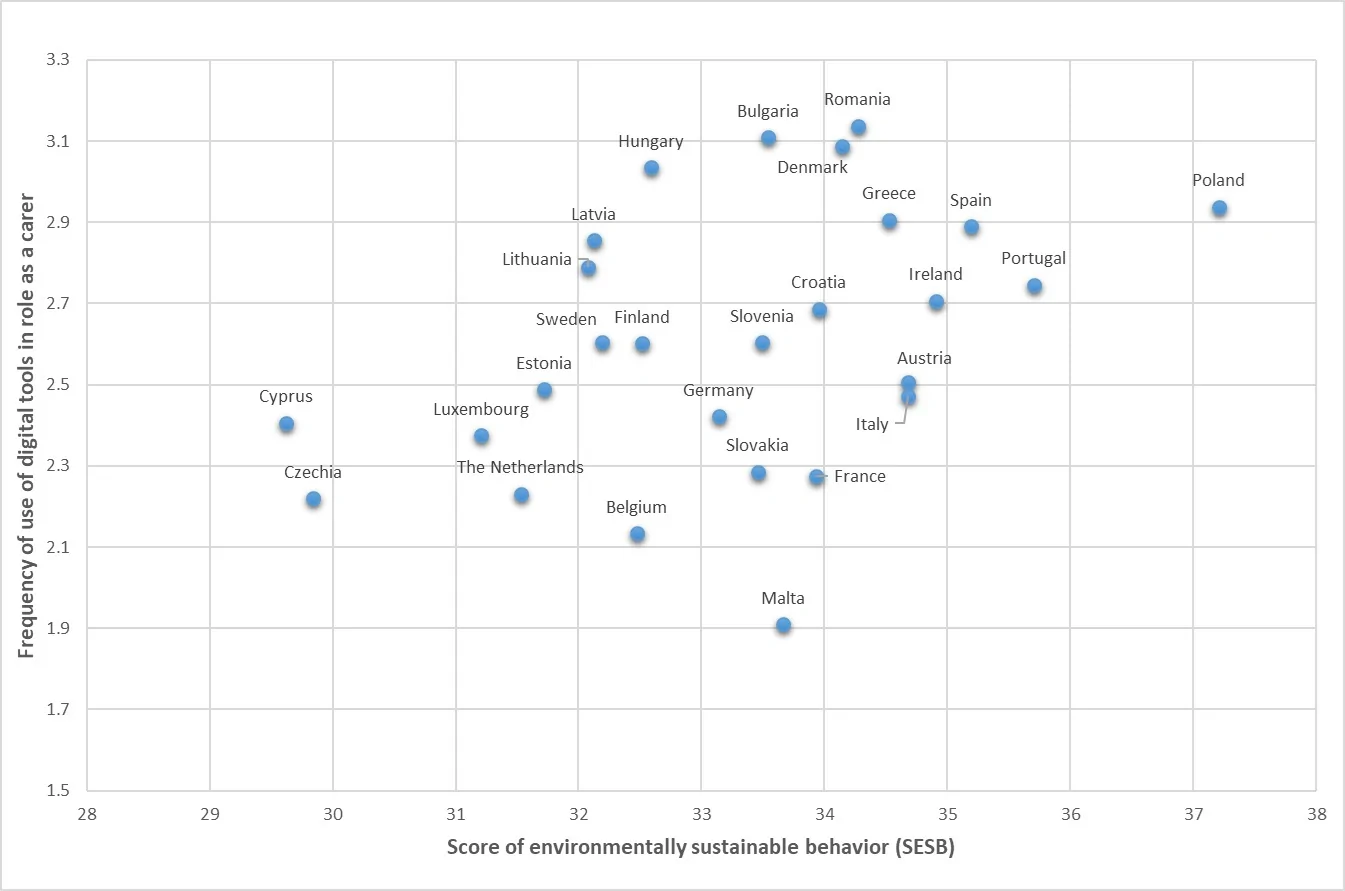
Frequency of use of digital tools and environmentally sustainable behaviors (in terms of score of environmentally sustainable behavior [SESB]) as a caregiver, by country.

The sociodemographic differences between users and never users raise the possibility of composition effects. To address this, a linear mixed effects model (maximum likelihood) was applied, fully adjusted for several potentially confounding variables: age, gender, educational level, weekly hours of informal care, degree of urbanization, perceived household income, working situation, residence of the care recipient, caregiver’s general health, and country (as a random effect). This adjustment controls for factors that could be associated with both the use of digital tools and environmentally sustainable behaviors. Figure 3 displays SESB across different frequencies of use of digital tools, showing higher SESB scores at higher frequencies of digital use. This is consistent with the bivariate analyses presented above.

**Figure 3.**
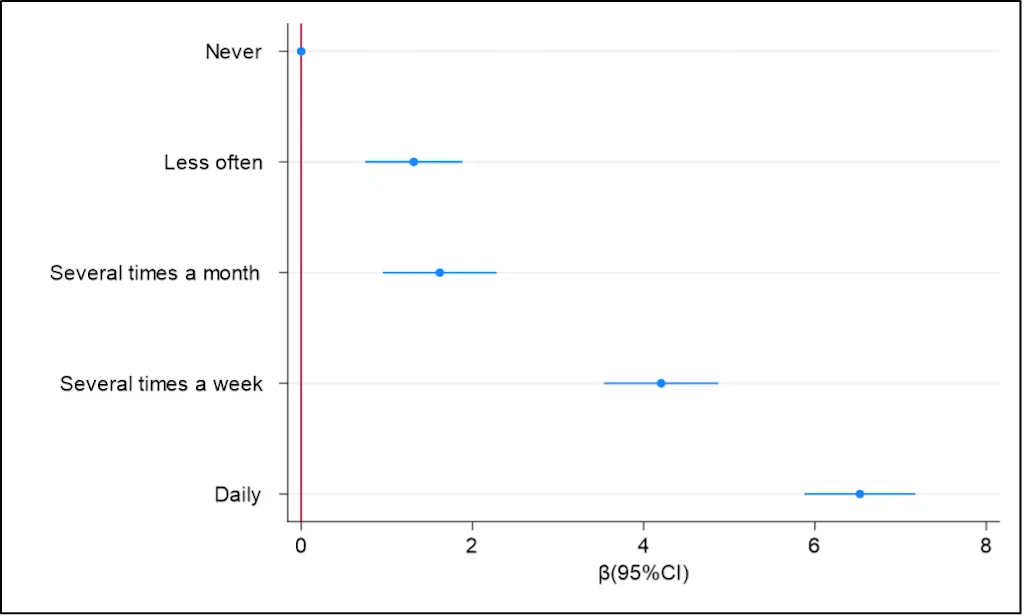
Forest plot: sustainable environmentally behavior score (SESB) according to the frequency of use of digital tools for the role of carer. Linear mixed effects (maximum likelihood) adjusted for age, gender, educational level, weekly hours of informal care for the main care recipient, degree of urbanization, perceived household income, working situation, residence of the main care recipient, caregiver’s general health, and country (random effect parameter).

The mixed effects ordered probit analyses were repeated for each of the items included in the SESB and are synthesized in Table 3. Like Figure 3, the models are fully adjusted for the same set of confounding variables and include a random effect for the country. Except for 3 eco-friendly behaviors—recycling, mindful consumption of natural resources, and prioritizing seasonal and local food products (for which statistical significance is present only for some of the frequencies of digital use)—statistical significance holds across all levels of use of digital tools, indicating that more frequent use of digital tools is associated with more frequent sustainable behaviors. This pattern is consistent with the previously observed peculiarity concerning behaviors like recycling and mindful consumption of natural resources.

**Table 3.** Environmentally sustainable behaviors according to the frequency of use of digital tools for the role of carer^a^.

Frequency of use of digital tools for role as carer	Avoidance of plastic and/or disposable/single-use products (Wald *χ*^2^_29_=314.00)		Recycling (Wald *χ*^2^_29_=187.39)		Avoidance of animal products (Wald *χ*^2^_29_=640.41)		Use of low-carbon-emission modes of transport (Wald *χ*^2^_29_=449.87)		Purchase of buy eco-friendly products and services (Wald *χ*^2^_29_=455.35)		Purchase of fair-trade products and services (Wald *χ*^2^_29_=588.23)		Purchase of used (instead of new) items (Wald *χ*^2^_29_=897.53)		Prioritize seasonal and local food products (Wald *χ*^2^_29_=300.84)		Mindful of recyclability or biodegradability of products and packaging (Wald *χ*^2^_29_=341.03)		Mindful of consumption of natural resources (Wald *χ*^2^_29_=277.62)	
	β (SE)	*P* value	β (SE)	*P* value	β (SE)	*P* value	β (SE)	*P* value	β (SE)	*P* value	β (SE)	*P* value	β (SE)	*P* value	β (SE)	*P* value	β (SE)	*P* value	β (SE)	*P* value
Ref. Never																				
Less often	0.16 (0.04)	<.001	–0.03 (0.04)	.55	0.16 (0.04)	<.001	0.19 (0.04)	<.001	0.15 (0.04)	<.001	0.23 (0.04)	<.001	0.23 (0.04)	<.001	0.03 (0.04)	.52	0.14 (0.04)	<.001	–0.08 (0.04)	.07
Several times a month	0.20 (0.05)	<.001	–0.20 (0.05)	<.001	0.37 (0.05)	<.001	0.14 (0.05)	.002	0.21 (0.05)	<.001	0.29 (0.05)	<.001	0.38 (0.05)	<.001	−0.03 (0.05)	.52	0.19 (0.05)	<.001	–0.24 (0.05)	<.001
Several times a week	0.43 (0.05)	<.001	–0.08 (0.05)	.10	0.57 (0.05)	<.001	0.38 (0.05)	<.001	0.47 (0.05)	<.001	0.57 (0.05)	<.001	0.62 (0.05)	<.001	0.21 (0.05)	<.001	0.40 (0.05)	<.001	–0.06 (0.05)	.22
Daily	0.60 (0.05)	<.001	0.16 (0.05)	.002	0.80 (0.05)	<.001	0.62 (0.05)	<.001	0.71 (0.05)	<.001	0.84 (0.05)	<.001	0.82 (0.05)	<.001	0.52 (0.05)	<.001	0.66 (0.05)	<.001	0.20 (0.05)	<.001

a Mixed effects probit regression adjusted for age, gender, educational level, weekly hours of informal care for the main care recipient, degree of urbanization, perceived household income, working situation, residence of the main care recipient, and caregiver’s general health, with county as a random effect.

From a practical perspective, this pattern suggests that a caregiver’s level of digital engagement is more than a technical characteristic; it may reflect a broader capacity to find information, compare alternatives, manage daily tasks, and access services.

## Discussion

### Principal Findings

This study provides large-scale empirical evidence that the use of digital tools among informal caregivers is positively associated with environmentally sustainable behaviors, expanding current understanding of how digital and ecological transitions intersect in LTC.

The main finding of a weak positive association [88] between the frequency of digital tools use and environmental behaviors (ρ=0.28; *P*<.001) in caregiving contexts provides empirical evidence that demographic, digital, and environmental transitions are interconnected rather than occurring in parallel. Extending the work by Tur-Sinai et al [68] on caregivers’ already heightened environmental engagement, our findings are consistent with the possibility that the use of digital tools is associated with caregivers’ preexisting environmental responsibility, an interpretation aligned with the ethics of care framework [70,71].

The comparatively larger associations with consumption-related behaviors suggest that improved access to information may be one possible pathway linking the use of digital tools to these behaviors, by helping caregivers identify, compare, and locate sustainable options. Digital platforms offer ways to identify eco-friendly products, compare fair-trade alternatives, locate second-hand goods, arrange delivery, or participate in online exchanges of reusable care-related items; these are the behaviors for which the associations were the largest. By contrast, the weaker associations with behaviors that depend on infrastructure (recycling, mindful resource consumption, and low-carbon transport) suggest that digital solutions alone may be insufficient: a caregiver may recognize the importance of recycling, but, without accessible municipal services, clear local instructions, or convenient collection points, digital engagement has limited practical leverage. The findings thus point to a differentiated intervention model, in which digital tools facilitate informed and convenient choices but must be complemented by physical infrastructure and social support where environmental action depends on local systems.

Furthermore, these behaviors are already widely established: 55% of respondents report engaging daily in recycling and mindful resource consumption, compared with 12%‐33% for other environmental behaviors. This suggests that recycling and resource conservation are long-established habits, likely formed before digital tools became widespread in caregiving. In line with previous research [82], such routine environmental behaviors tend to be less responsive to digital engagement, as they are embedded in habit rather than informed choice. By contrast, consumption-related behaviors—such as buying secondhand or ecolabeled products—are more variable and may be more closely associated with informational and motivational inputs enabled through digital tools [48,51,53]. Consequently, these behaviors show larger associations with digital engagement.

These results support the distinction drawn in the literature between habitual and intentional pro-environmental actions [89], while extending it to the specific context of informal caregiving. In doing so, they offer both theoretical insights and practical guidance for integrated action across the domains of care, digital inclusion, and sustainability. Overall, the observed associations between the use of digital tools and eco-friendly behaviors may operate through attitudes, subjective norms, and perceived behavioral control, although these mechanisms were not directly tested. Digital access to information about sustainable products [48,49] may inform caregivers’ evaluative judgments, particularly for consumption choices requiring informed decision-making. Online shopping may support perceived behavioral control by making sustainable choices more accessible [53], especially valuable for caregivers who cannot leave care recipients needing round-the-clock assistance. Online caregiver communities, where sustainable practices are shared and encouraged [56,62], may also relate to subjective norms about what is considered normal or expected. Regarding demographic patterns, consistent with established research on digital divides [66], the adoption of digital tools shows systematic variation across demographic groups, with higher rates among younger, more educated, and employed caregivers. Although the education gap is not substantial and the urban-rural divide is moderate, these disparities suggest that digital-environmental co-occurrence may be unevenly distributed. Older, less educated, and nonemployed caregivers—often those providing the most intensive care—appear less likely to use digital tools frequently. This may leave existing inequities unaddressed unless digital inclusion strategies specifically target these groups [66,67].

From a SEIS framework perspective [31], these findings demonstrate how the infrastructural system (digital tools) might shape behaviors within the ecological system through social system mediators (caregiver decision-making processes).

### Policy and Practice Implications

First, policy frameworks addressing demographic, digital, and environmental transitions in LTC should recognize their interconnections rather than treating them as separate domains. Integrated approaches that simultaneously address caregiving needs, digital inclusion, and environmental sustainability could bring collateral benefits across multiple transition domains.

Second, to avoid exacerbating existing inequalities in the care sector [1,8], digital inclusion initiatives should specifically target more vulnerable caregiver groups or those facing greater barriers—particularly older, less educated, and nonemployed caregivers—to ensure that the benefits of digital-environmental synergies are equitably distributed across the caregiver population.

Third, environmental policies and LTC service providers could consider caregivers’ existing predisposition toward environmental responsibility [68] when designing digital tools with dual functions, so that the same platforms supporting caregiving tasks could also provide access to sustainable consumption choices, particularly for those providing intensive care who may have limited mobility or greater time constraints. For example, existing caregiver support apps or portals could incorporate features such as mapping locally available second-hand or fair-trade suppliers and low-emission transport options for accompanying care recipients to appointments. Since the findings show a larger association with consumption-related behaviors, integrating sustainable-procurement guidance directly into the tools caregivers already use may be a relevant entry point.

Finally, educational programs for caregivers should integrate digital literacy with environmental awareness, helping caregivers understand how digital tools can facilitate both caregiving responsibilities and environmentally sustainable practices.

These recommendations must also take into account that family caregivers already perform demanding tasks that generate substantial waste. Expecting them to adopt greener practices without structural support risks adding further burden. Since caregivers already lean toward sustainability, the priority is not to demand more of them but to make sustainable action easier through the provision of efficient recyclable products and accessible disposal systems. Digital tools are most valuable here when they reduce, rather than add to, the effort of acting sustainably.

Overall, these results highlight the importance of considering all 3 SEIS dimensions when designing interventions to support the triple transition in LTC. Importantly, digital tools cannot substitute for physical infrastructure. Their associations are the largest for behaviors individuals can act on directly (such as purchasing choices) and weakest where action depends on external provision, such as recycling and resource conservation. The observed associations therefore suggest that the environmental relevance of digital investments may depend on whether enabling infrastructure already exists. Policies should combine digital inclusion measures with complementary local infrastructure, such as low-carbon transport or accessible recycling facilities, so that the options digital platforms recommend are available to caregivers. Digital innovation and physical infrastructure should be considered coordinated investments rather than alternatives.

### Limitations

Although our study provides important insights into the intersection of the use of digital tools and environmental behaviors among informal caregivers, 2 primary limitations should be acknowledged. First, the cross-sectional nature of our data does not allow us to establish causality or temporal ordering in the relationship between the use of digital tools and environmental behaviors. The mechanisms discussed in this paper should therefore be understood as theory-guided and empirically plausible pathways rather than as directly observed causal relationships. Nevertheless, the differentiated pattern across behavior categories offers useful indirect evidence: larger associations for information- and consumption-related behaviors and weaker associations for infrastructure-dependent behaviors are consistent with the proposed TPB and SEIS interpretation. Second, our measurement of digital tools captured frequency rather than the specific type, content, or function of the tools. As a result, the study cannot directly test whether caregivers used digital tools for environmental information, online purchasing, care coordination, peer support, or service navigation, which limits our understanding of which digital applications are most closely associated with eco-friendly behaviors. In light of these limitations, further mixed method studies are encouraged—combining longitudinal data with semistructured interviews—to clarify the temporal ordering and direction of the relationship between digital tools and environmental behaviors, to identify which types of tools caregivers use in relation to eco-friendly practices, and to examine the determinants and motivations underlying environmental attitudes. A further limitation derives from the CAWI recruitment procedure. Because the CAWI sample was drawn from online access panels, participants in these countries had baseline internet access and were registered with an online panel. Consequently, the “never users of digital tools for caregiving” group should not be interpreted as equivalent to noninternet users in the general population. The present dataset therefore cannot provide evidence about caregivers who were offline or otherwise excluded from online access panels, and claims concerning digital exclusion should be interpreted accordingly. Although the sample was weighted by country according to sex, age, region, and educational level, weighting cannot remove this underlying coverage limitation. Despite these limitations, this study provides empirical evidence of the associations between the use of digital tools and environmental behaviors among informal caregivers, contributing to our understanding of how the triple transition manifests in everyday caregiving contexts.

### Conclusions

This study provides empirical evidence of an association between the use of digital tools and environmental practices among informal caregivers across the EU, suggesting that demographic, digital, and environmental transitions may co-occur rather than operate in isolation. However, because the design is cross-sectional, these findings should be read as associations rather than as evidence that the use of digital tools changes environmental behavior, and they may not generalize to caregivers without baseline internet access. Overall, the findings support integrated approaches that combine digital inclusion, caregiver support, and environmental infrastructure, while recognizing that the strength of the observed associations varies across behavior types and that recycling and resource conservation were not significantly correlated with the use of digital tools. However, these potential benefits might not be equally accessible to all caregivers, increasing the risk that digital tools may inadvertently widen existing inequalities in the care sector. Realizing the full potential of digital tools to support both caregiving and environmental goals will require inclusive access and appropriate infrastructure support. In practical terms, digitalization in LTC should be valued not only for improving care efficiency but also for its potential to support caregivers’ environmental practices by reducing the burden of acting sustainably. This potential is behavior-specific: digital tools are most likely to help with information-, purchasing-, and coordination-related behaviors and least likely to help with practices constrained by physical infrastructure or established routines.

As European countries continue to face the triple transition, the evidence presented in this paper provides a foundation for developing more integrated approaches that combine sustainability-oriented digital platforms, caregiver training in both digital and environmental literacy, and the local infrastructure needed to translate digital information into feasible action.

## Supplementary material

10.2196/81556 Multimedia Appendix 1 Supplementary 1: ten questions list of the score of environmentally sustainable behavior (SESB) score.

## Abbreviations

CATI: computer-assisted telephone interview
CAWI: computer-assisted web interview
EIGE: European Institute for Gender Equality
EU: European Union
GDPR: General Data Protection Regulation
INRCA: Istituto Nazionale Ricovero e Cura Anziani
IRCCS: Istituto di Ricovero e Cura a Carattere Scientifico
ISCED: International Standard Classification of Education
LTC: long-term care
NUTS III: Nomenclature of Territorial Units for Statistics–Level 3
OECD: Organisation for Economic Co-operation and Development
SEIS: social-ecological-infrastructural systems
SESB: score of environmentally sustainable behavior
TPB: theory of planned behavior

## References

[1] (2023). Towards a Triple Transition: Strategies for Transformational European Development Action. https://www.oecd.org/content/dam/oecd/en/publications/reports/2023/10/towards-a-triple-transition_0e9ebd19/094322ba-en.pdf.

[2] Eurostat Population structure indicators at national level. https://doi.org/10.2908/DEMO_PJANIND.

[3] Eurofound (2025). Unpaid care in the EU. https://assets.eurofound.europa.eu/f/279033/16470d0bab/ef25001en.pdf.

[4] Rocard E, Nozal A (2022). Supporting informal carers of older people: policies to leave no carer behind. https://www.oecd.org/content/dam/oecd/en/publications/reports/2022/05/supporting-informal-carers-of-older-people_447892d0/0f0c0d52-en.pdf.

[5] Atasoy H, Chen P yu, Ganju K (2018). The spillover effects of health IT investments on regional health care costs. Manage Sci.

[6] Fragão-Marques M, Ozben T (2023). Digital transformation and sustainability in healthcare and clinical laboratories. Clin Chem Lab Med.

[7] Wanka A, Urbaniak A, Oswald F, Kolland F (2023). Digital transformations in ageing societies. Z Gerontol Geriat.

[8] Choudrie J, Zamani E, Obuekwe C (2022). Bridging the digital divide in ethnic minority older adults: an organisational qualitative study. Inf Syst Front.

[9] Watts N, Amann M, Arnell N (2019). The 2019 report of The Lancet Countdown on health and climate change: ensuring that the health of a child born today is not defined by a changing climate. The Lancet.

[10] WHO (2020). Operational framework for building climate resilient and environmentally sustainable health care facilities. https://iris.who.int/server/api/core/bitstreams/9807d810-5279-4e26-886c-3c98e6b992d0/content.

[11] Braithwaite J, Ludlow K, Testa L (2020). Built to last? The sustainability of healthcare system improvements, programmes and interventions: a systematic integrative review. BMJ Open.

[12] Spasova S, Baeten R, Vanhercke B, European Commission: Directorate-General for Employment, Social Affairs and Inclusion (2018). Challenges in long-term care in Europe.

[13] WHO (2021). Global Strategy on Digital Health 2020-2025.

[14] Kruse CS, Mileski M, Vijaykumar AG, Viswanathan SV, Suskandla U, Chidambaram Y (2017). Impact of electronic health records on long-term care facilities: systematic review. JMIR Med Inform.

[15] Lyons KS, Zarit SH, Sayer AG, Whitlatch CJ (2002). Caregiving as a dyadic process: perspectives from caregiver and receiver. J Gerontol B Psychol Sci Soc Sci.

[16] Wright J, Hamblin K, Hamblin K, Lariviere M (2023). Care Technologies for Ageing Societies.

[17] Yeung AWK, Torkamani A, Butte AJ (2023). The promise of digital healthcare technologies. Front Public Health.

[18] Hamblin KA (2022). Technology in care systems: displacing, reshaping, reinstating or degrading roles?. New Technol Work Employ.

[19] Moyle W, Jones C, Dwan T, Ownsworth T, Sung B (2019). Using telepresence for social connection: views of older people with dementia, families, and health professionals from a mixed methods pilot study. Aging Ment Health.

[20] Willard S, Cremers G, Man YP, van Rossum E, Spreeuwenberg M, de Witte L (2018). Development and testing of an online community care platform for frail older adults in the Netherlands: a user-centred design. BMC Geriatr.

[21] Pereno A, Eriksson D (2020). A multi-stakeholder perspective on sustainable healthcare: from 2030 onwards. Futures.

[22] Muschol J, Heinrich M, Heiss C (2022). Economic and environmental impact of digital health app video consultations in follow-up care for patients in orthopedic and trauma surgery in Germany: randomized controlled trial. J Med Internet Res.

[23] Greenhalgh T, Wherton J, Papoutsi C (2017). Beyond adoption: a new framework for theorizing and evaluating nonadoption, abandonment, and challenges to the scale-up, spread, and sustainability of health and care technologies. J Med Internet Res.

[24] Castle-Clarke S (2018). What will new technology mean for the NHS and its patients?. https://assets.kingsfund.org.uk/f/256914/x/0158561602/nhs_70_what_will_new_technology_mean_2018.pdf.

[25] Mosca O, Manunza A, Manca S, Vivanet G, Fornara F (2023). Digital technologies for behavioral change in sustainability domains: a systematic mapping review. Front Psychol.

[26] Melkas H, Hennala L, Pekkarinen S, Kyrki V (2020). Impacts of robot implementation on care personnel and clients in elderly-care institutions. Int J Med Inform.

[27] Tarpani RRZ, Gallego-Schmid A (2024). Environmental impacts of a digital health and well-being service in elderly living schemes. Clean Environ Syst.

[28] Alami H, Rivard L, Lehoux P, Ag Ahmed MA, Fortin JP, Fleet R (2023). Integrating environmental considerations in digital health technology assessment and procurement: stakeholders’ perspectives. Digit Health.

[29] Pekkarinen S, Melkas H, Hyypiä M, Toivonen M, Saari E (2019). Human-Centered Digitalization and Services.

[30] EAN (2024). Accelerating the digital transition: A stepping stone for sustainable elderly care in Europe. https://www.ean.care/media/fileman/2024_EAN_Accelerating_the_digital.pdf.

[31] Ramaswami A, Weible C, Main D (2012). A social‐ecological‐infrastructural systems framework for interdisciplinary study of sustainable city systems. J Industrial Ecology.

[32] Ostrom E (2009). A general framework for analyzing sustainability of social-ecological systems. Science.

[33] McPhearson T, Pickett STA, Grimm NB (2016). Advancing urban ecology toward a science of cities. Bioscience.

[34] Pickett STA, Cadenasso ML, Grove JM (2011). Urban ecological systems: scientific foundations and a decade of progress. J Environ Manage.

[35] Boone CG, Cook E, Hall SJ (2012). A comparative gradient approach as a tool for understanding and managing urban ecosystems. Urban Ecosyst.

[36] Grimm NB, Cook EM, Hale RL, Iwaniec DM, Seto KC, Solecki W, Griffith CA (2015). The Routledge Handbook of Urbanization and Global Environmental Change.

[37] Meerow S, Newell JP (2019). Urban resilience for whom, what, when, where, and why?. Urban Geogr.

[38] Krueger EH, Constantino SM, Centeno MA, Elmqvist T, Weber EU, Levin SA (2022). Governing sustainable transformations of urban social-ecological-technological systems. NPJ Urban Sustain.

[39] Alshaikh RZ, Shok ME, Al-Hussaini ZIH, Alkinani AS (2024). The impact of digital technologies on urban life quality and social dynamics in bismayah. Int J Sustain Dev Plan.

[40] Purvis B, Mao Y, Robinson D (2019). Three pillars of sustainability: in search of conceptual origins. Sustain Sci.

[41] Pricopoaia O, Cristache N, Lupașc A, Iancu D (2025). The implications of digital transformation and environmental innovation for sustainability. J Innov Knowl.

[42] Chopra R, Agrawal A, Sharma GD, Kallmuenzer A, Vasa L (2024). Uncovering the organizational, environmental, and socio-economic sustainability of digitization: evidence from existing research. Rev Manag Sci.

[43] Thøgersen J (1999). Spillover processes in the development of a sustainable consumption pattern. J Econ Psychol.

[44] Hilty LM, Aebischer B, Hilty LM, Aebischer B (2015). ICT Innovations for Sustainability.

[45] Degavre F, Kieffer S, Bol D (2022). Searching for sustainability in health systems: toward a multidisciplinary evaluation of mobile health innovations. Sustainability.

[46] Kwon C, Essayei L, Spencer M (2024). The environmental impacts of electronic medical rcords versus paper records at a large eye hospital in India: life cycle assessment study. J Med Internet Res.

[47] Thompson M (2021). The environmentally impacts of digital health. Digit Health.

[48] Santarius T, Pohl J, Lange S (2020). Digitalization and the decoupling debate: can ICT help to reduce environmental impacts while the economy keeps growing?. Sustainability.

[49] Johnson D, Horton E, Mulcahy R, Foth M (2017). Gamification and serious games within the domain of domestic energy consumption: a systematic review. Renew Sust Energ Rev.

[50] Štreimikienė D, Baležentis A (2015). Assessment of willingness to pay for renewables in Lithuanian households. Clean Techn Environ Policy.

[51] Islam MS, Proma AM, Wohn C (2023). SEER: sustainable e-commerce with environmental-impact rating. Clean Environ Syst.

[52] Senturk G, Dumludag D (2022). The relationship between consumption of single-use plastic bags, environmental awareness, and socio-demographic factors. J Mater Cycles Waste Manag.

[53] Eunike IJ, Silalahi ADK, Phuong DTT, Tedjakusuma AP (2025). Towards a holistic model of green consumerism by integrating ethical obligations, collective efficacy, and environmental concerns in sustainable purchase behavior. Sustainable Futures.

[54] Rana S, Shafi F, Rasheed A, Malik MI (2024). Online environmental platforms service and green consumer behavior nexus: a multi-mediator study. Futur Bus J.

[55] Geiger N, Swim JK, Glenna L (2019). Spread the green word: a social community perspective into environmentally sustainable behavior. Environ Behav.

[56] Hynes N, Wilson J (2016). I do it, but don’t tell anyone! Personal values, personal and social norms: Can social media play a role in changing pro-environmental behaviours?. Technol Forecast Soc Change.

[57] Kakulla B (2023). 2023 tech trends: no end in sight for age 50+ market growth. AARP Research.

[58] Keenan TA (2022). Using technology to assist with caregiving: caregivers are interested. AARP Research.

[59] Hassan AYI (2020). Challenges and recommendations for the deployment of information and communication technology solutions for informal caregivers: scoping review. JMIR Aging.

[60] Soares S, Hoffmeister LV, Fernandes M de F, Henriques A, Costa A (2024). The use of digital technologies in the promotion of health literacy and empowerment of informal caregivers: scoping review. JMIR Aging.

[61] Chi NC, Demiris G (2015). A systematic review of telehealth tools and interventions to support family caregivers. J Telemed Telecare.

[62] Wallcook S, Nygård L, Kottorp A, Malinowsky C (2021). The use of everyday information communication technologies in the lives of older adults living with and without dementia in Sweden. Assist Technol.

[63] Yin Z, Stratton L, Song Q (2024). Perceptions and utilization of online peer support among informal dementia caregivers: survey study. JMIR Aging.

[64] Guay C, Auger C, Demers L (2017). Components and outcomes of internet-based interventions for caregivers of older adults: systematic review. J Med Internet Res.

[65] Czaja SJ, Perdomo D, Lee CC (2016). Human Aspects of IT for the Aged Population Design for Aging.

[66] Shaffer KM, Chow PI, Cohn WF, Ingersoll KS, Ritterband LM (2018). Informal caregivers’ use of internet-based health resources: an analysis of the health information national trends survey. JMIR Aging.

[67] Schütz J (2023). Who benefits from digital transformation in (health) care? Internet use among informal carers and care recipients in Europe.

[68] Tur-Sinai A, Bentur N, Lamura G, Rodrigues R, Di Rosa M, Socci M (2025). The sustainability spillover: uncovering the link between informal elder care and eco-conscious behaviors across the European Union. Innov Aging.

[69] Cousins EM (2021). Mobilizing motherhood: the gendered burden of environmental protection. Sociol Compass.

[70] Gilligan C (1982). In a Different Voice: Psychological Theory and Women’s Development.

[71] Tronto J (2020). Moral Boundaries: A Political Argument for an Ethic of Care.

[72] Xiao C, Hong D (2010). Gender differences in environmental behaviors in China. Popul Environ.

[73] Migheli M (2021). Green purchasing: the effect of parenthood and gender. Environ Dev Sustain.

[74] Pinquart M, Sörensen S (2007). Correlates of physical health of informal caregivers: a meta-analysis. J Gerontol B Psychol Sci Soc Sci.

[75] Schulz R, Beach SR, Matthews JT, Courtney K, De Vito Dabbs A, Mecca LP (2016). Caregivers’ willingness to pay for technologies to support caregiving. Gerontologist.

[76] Wagle S, Yang S, Osei EA, Katare B, Lalani N (2024). Caregiving intensity, duration, and subjective financial well-being among rural informal caregivers of older adults with chronic illnesses or disabilities. Health Care (Don Mills).

[77] Spann A, Vicente J, Allard C, Hawley M, Spreeuwenberg M, de Witte L (2020). Challenges of combining work and unpaid care, and solutions: a scoping review. Health Soc Care Community.

[78] Lutz C, Hoffmann CP, Ranzini G (2020). Data capitalism and the user: an exploration of privacy cynicism in Germany. New Media Soc.

[79] Greenfield EA, Oberlink M, Scharlach AE, Neal MB, Stafford PB (2015). Age-friendly community initiatives: conceptual issues and key questions. Gerontologist.

[80] de Leeuw A, Valois P, Ajzen I, Schmidt P (2015). Using the theory of planned behavior to identify key beliefs underlying pro-environmental behavior in high-school students: Implications for educational interventions. J Environ Psychol.

[81] Armitage CJ, Conner M (2001). Efficacy of the theory of planned behaviour: a meta-analytic review. Br J Soc Psychol.

[82] Stern PC (2000). New environmental theories: toward a coherent theory of environmentally significant behavior. J Soc Issues.

[83] EIGE (2023). Online panel survey on gender gaps in unpaid care, individual and social activities - technical report.

[84] Austin PC, Goel V, van Walraven C (2001). An introduction to multilevel regression models. Can J Public Health.

[85] Hox J, Balderjahn J, Mathar R, Schader M (1998). Classification, Data Analysis, and Data Highways Studies in Classification, Data Analysis, and Knowledge Organization.

[86] (2023). European institute for gender equality (EIGE). survey of gender gaps in unpaid CARE, individual and social activities (CARE) (ZA8752; version 1.0.0). GESIS.

[87] Global Privacy & Data Protection Policy. IPSOS.

[88] Cohen J (1988). Statistical Power Analysis for the Behavioral Sciences.

[89] Kurz T, Gardner B, Verplanken B, Abraham C (2015). Habitual behaviors or patterns of practice? Explaining and changing repetitive climate‐relevant actions. WIREs Climate Change.

