# Supplementary material for: Use of Digital Tools and Environmental Practices Among Informal Caregivers of Older Adults in the European Union Embracing the Triple Transition: Cross-Sectional Study
Source: JMIR Aging. 2026 Nov 13;9:e81556. doi: 10.2196/81556 (PMC13766587; doi:10.2196/81556)
Supplement: Supplementary file 1 [file aging-v9-e81556-s001.docx]

**Supplementary Material 2 - Appendix**

**Ten questions list of the SESB score**

Outcome variable of the study was a score calculated from participants’ answers to the following questions, referring to environmentally friendly behaviors: “There is much debate today about the impact of human society on the global environment. Generally thinking about your personal consumption and behavior, how often.…:

1. “do you avoid plastic and/or disposable/single-use products?
2. “do you recycle?
3. “do you avoid animal products?
4. “are you using low carbon-emission modes of transport (cycling, public transport, car-pooling instead of your personal car)?
5. do you buy sustainable or eco-friendly products and services?
6. do you buy fair-trade products and services?
7. do you buy used instead of new items (e.g., second-hand clothes, used furniture)?
8. do you prioritize seasonal and local food products?
9. do you pay attention that products or packaging are recyclable or biodegradable?
10. are you mindful of your consumption of natural resources (water, electricity and heating)?

Potential responses to the above were: daily; several times weekly; several times monthly; less often; never.

To build the Score of Environmentally Sustainable Behavior (SESB), the questions were coded as: 1=“Never”; 2=“Less often”; 3=“Several times a month”; 4=“Several times a week”; 5= “Daily.” Then, they were summed in order to obtain a score from 10 to 50 (the higher the score, the higher the respondent’s environmental behavior).

The “frequency of use of digital tools in role as a carer for people who depend on help with daily living activities (e.g. E-health tools, web platforms / apps with reminders about medications)” variable was coded as follows: 1=“Never”; 2=“Less often”; 3=“Several times a month”; 4=“Several times a week”; 5= “Daily.”
